# Clinical Outcomes After Percutaneous Coronary Intervention in Patients With Cirrhosis: A Multicentre National Study

**DOI:** 10.1111/apt.70896

**Published:** 2026-08-05

**Authors:** Dhir Gala, Sameer Rao, Manas Gunani, Kajol Shah, Anand V. Kulkarni, Karn Wijarnpreecha, Daniel Q. Huang, Pojsakorn Danpanichkul, Ahmed Al‐Khazraji, Kaveh Hajifathalian, Vivek Lingiah, Paul Gaglio, Nikki Duong, Christine Gerula

**Affiliations:** ^1^ Department of Medicine Rutgers New Jersey Medical School Newark New Jersey USA; ^2^ Allegheny Health Network Pittsburgh Pennsylvania USA; ^3^ Divison of Cardiology Rutgers New Jersey Medical School Newark New Jersey USA; ^4^ Division of Gastroenterology and Hepatology Asian Institute of Gastroenterology Hyderabad India; ^5^ Division of Gastroenterology and Hepatology University of Arizona College of Medicine Phoenix Arizona USA; ^6^ Division of Gastroenterology and Hepatology National University of Singapore Singapore Singapore; ^7^ Department of Medicine Texas Tech University Health Science Center Lubbock Texas USA; ^8^ Division of Gastroenterology and Hepatology Rutgers New Jersey Medical School Newark New Jersey USA; ^9^ Division of Gastroenterology and Hepatology Stanford University Stanford California USA

**Keywords:** antiplatelet therapy, cirrhosis, coronary artery disease, percutaneous coronary intervention

## Abstract

**Background:**

Coronary artery disease (CAD) is common in cirrhosis patients because of shared risk factors, including metabolic syndrome and alcohol use; however, outcomes after percutaneous coronary intervention (PCI) remain poorly defined.

**Aims:**

To evaluate outcomes after PCI in patients with cirrhosis.

**Methods:**

We conducted a retrospective cohort study using TriNetX US Collaborative Network data from 2015 to 2024. Adults undergoing PCI were stratified by cirrhosis status, with outcomes including gastrointestinal bleeding, ischaemic events, and all‐cause mortality. Within the cirrhosis cohort, we evaluated effect modification by aetiology, disease severity, and antiplatelet strategy at 30 days after PCI. One‐to‐one propensity score matching adjusted for demographics and comorbidities, with results reported as relative risks and 95% confidence intervals.

**Results:**

Among 3351 patients with cirrhosis undergoing PCI matched 1:1 to controls, cirrhosis was associated with higher 1‐year gastrointestinal bleeding (16.14% vs. 8.12%; *p* < 0.0001) and mortality (13.97% vs. 10.41%; *p* < 0.0001). Decompensated cirrhosis had higher gastrointestinal bleeding and mortality than compensated disease, while alcohol‐associated cirrhosis had higher gastrointestinal bleeding than metabolic dysfunction‐associated steatotic liver disease (MASLD) cirrhosis. In a 30‐day landmark analysis, antiplatelet monotherapy (aspirin or P2Y12 inhibitors) had lower 1‐year gastrointestinal bleeding (10.31% vs. 20.77%; *p* < 0.0001) and mortality (9.46% vs. 12.89%; *p* = 0.04) without increased stent restenosis compared with dual antiplatelet therapy (DAPT).

**Conclusion:**

Cirrhosis confers substantially higher bleeding and mortality after PCI, particularly in decompensated disease. Antiplatelet monotherapy after 30 days was associated with less bleeding and improved survival, supporting abbreviated DAPT in this high‐risk group.

## Graphical abstract

Summary of a multicentre TriNetX study evaluating outcomes after percutaneous coronary intervention (PCI) in patients with cirrhosis. Cirrhosis was associated with higher 1‐year gastrointestinal bleeding and mortality, especially with decompensated disease. Antiplatelet monotherapy after 30 days was associated with lower bleeding and mortality than dual antiplatelet therapy.

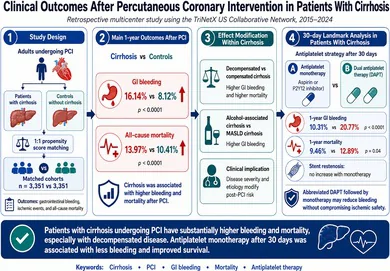

## 1 Introduction

Cirrhosis has emerged as a growing challenge in cardiovascular medicine, as the increasing prevalence of both chronic liver disease and coronary artery disease has created a complex patient population requiring coronary revascularisation [1]. In the United States, cirrhosis is projected to affect approximately 2.5 million individuals by 2030, primarily driven by the growing burden of metabolic dysfunction–associated steatotic liver disease (MASLD) [2, 3, 4, 5]. MASLD currently affects nearly one‐third of the adult USA population and has become the second leading indication for liver transplantation nationally, as well as a significant global cause of cirrhosis‐related mortality [6, 7, 8, 9].

Coronary artery disease (CAD) is estimated to affect approximately one in five patients with cirrhosis [10, 11]. Although cirrhosis itself may not be an independent risk factor for CAD, the cardiovascular burden in this population appears to be primarily driven by shared cardiometabolic risk factors, including hypertension, diabetes mellitus, and hyperlipidemia [10, 12, 13]. Notably, CAD prevalence varies by cirrhosis aetiology, with the highest rates observed in MASLD‐related cirrhosis (21%) and alcohol‐associated cirrhosis (17%), compared with substantially lower rates in primary biliary cholangitis (4%) and viral hepatitis–related cirrhosis (5%) [10]. In addition, CAD is also a major cause of both early and late post‐liver transplant complications, including perioperative myocardial infarction, heart failure, and mortality. Therefore, the accurate identification of CAD is an integral part of the patient work‐up before liver transplant [14, 15].

The high prevalence of CAD in cirrhosis patients has led to an increase in patients with cirrhosis requiring revascularisation, either in the setting of acute ischemia or chronic CAD, especially during pre‐transplant workup [16, 17]. Percutaneous coronary intervention (PCI) has become the preferred revascularisation strategy for patients with cirrhosis and CAD because coronary artery bypass grafting is associated with high mortality in this population, particularly in patients with portal hypertension [18, 19]. However, the coexistence of PCI and cirrhosis presents a clinical dilemma. Currently, data and guideline recommendations regarding PCI outcomes in patients with cirrhosis remain limited, as most landmark PCI trials have excluded individuals with cirrhosis or hepatic dysfunction, and existing studies are constrained by small sample sizes or single‐centre designs [20]. Observational studies using the National Inpatient Sample have demonstrated high post‐PCI in‐hospital mortality among patients with cirrhosis, with 90‐day mortality reaching 10.3% compared with 2.5% in patients without liver disease [21, 22]. Despite these findings, important knowledge gaps persist regarding long‐term outcomes beyond the index hospitalisation, the optimal duration and composition of antiplatelet therapy, and whether outcomes differ by the underlying aetiology of cirrhosis.

A major challenge in post‐PCI management among patients with cirrhosis is determining the most appropriate dual antiplatelet therapy (DAPT) strategy. Although DAPT with aspirin and a P2Y12 inhibitor is the standard of care after coronary stenting and reduces recurrent ischaemic events in the general population, patients with cirrhosis face a substantially higher risk of gastrointestinal bleeding due to coagulopathy and manifestations of portal hypertension including portal gastropathy, oesophageal and gastric varices, thrombocytopenia, and platelet dysfunction [23, 24]. Multiple randomised controlled trials support shorter durations of dual antiplatelet therapy (DAPT) of 1–3 months followed by P2Y12 inhibitor monotherapy to reduce bleeding without compromising ischaemic protection, particularly in high bleeding risk populations [25, 26]. However, patients with cirrhosis have been excluded from these studies, leaving no comparative data to guide DAPT duration, long‐term antiplatelet strategy, or selection of monotherapy in this population. As a result, clinicians must empirically balance the risk of gastrointestinal bleeding associated with prolonged DAPT against ischaemic complications such as stent thrombosis or recurrent myocardial infarction with abbreviated DAPT regimens. To address these gaps, we conducted a retrospective multicentre cohort study of adults undergoing PCI to compare bleeding events, ischaemic complications, and mortality in patients with and without cirrhosis.

## 2 Methods

### 2.1 Database

TriNetX is a federated research platform that provides access to deidentified electronic health record data from healthcare organisations in the United States and internationally. This study utilised data from the United States Collaborative Network, comprising 72 participating sites within the United States. The database contains longitudinal information on patient demographics, clinical diagnoses, medical procedures, prescription medications, laboratory test results, and mortality outcomes. Data elements are standardised using established clinical coding systems, including the International Classification of Diseases (ICD), Current Procedural Terminology (CPT), Logical Observation Identifiers Names and Codes (LOINC), and RxNorm standardised nomenclature for clinical drugs (RxNorm). Analyses are conducted within secure institutional environments, with only aggregated, nonidentifiable results returned to investigators [27].

### 2.2 Study Design

We conducted a retrospective cohort study of adults in the United States using the TriNetX research network between 1 January 2015, and 31 December 2024. Institutional review board approval was not required because the study used deidentified data.

We identified patients who underwent percutaneous coronary intervention (PCI) using ICD‐10 codes for stent placement, atherectomy, or both; procedures limited to balloon angioplasty were excluded to avoid heterogeneity in post‐procedural management and antiplatelet therapy (Table S1). Patients with prior coronary artery bypass grafting or liver transplantation were excluded to avoid confounding related to advanced cardiovascular or liver disease states. Cohorts were defined by the presence or absence of cirrhosis and matched 1:1 using propensity scores based on demographics, comorbidities, and potential indications for PCI. Covariates were captured during the 1‐year period before and including the date of PCI.

Outcomes were assessed at 30 days and 1 year after PCI and included cardiovascular, Haemorrhagic, thrombotic, neurological, renal, healthcare utilisation outcomes, and all‐cause mortality. Recurrent myocardial infarction and repeat PCI were not reported as outcomes because prior coding limited reliable identification of incident events.

We also performed an exploratory analysis within the cirrhosis cohort to identify clinical factors associated with 1‐year all‐cause mortality after PCI. The variables evaluated included demographics, cirrhosis aetiology, hepatic decompensation events, PCI indication, and laboratory measures reflecting hepatic and renal function.

### 2.3 Sub‐Group Analyses

Prespecified subgroup analyses were performed within the cirrhosis cohort to explore effect modification by cirrhosis aetiology, disease severity, and post‐PCI antiplatelet strategy. For each subgroup, cohorts were compared after propensity score matching using the same clinically relevant variables as in the primary analysis. Cirrhosis aetiology was additionally included in the propensity model for all subgroup analyses except the comparison between alcohol‐associated cirrhosis and MASLD cirrhosis. Laboratory parameters reflecting liver disease severity were also incorporated, except in the comparison of compensated versus decompensated cirrhosis.

For aetiology‐based comparisons, patients with chronic hepatitis B or C were excluded. MASLD cirrhosis was defined as cirrhosis with at least one metabolic risk factor and no alcohol‐associated liver disease, whereas alcohol‐associated cirrhosis was defined as cirrhosis attributed to alcohol use without concurrent metabolic dysfunction; patients with metabolic dysfunction–associated alcohol‐related liver disease (MetALD) were excluded.

Cirrhosis severity was categorised based on clinical decompensation, with decompensated cirrhosis defined by a history of ascites, portal hypertension, variceal bleeding, hepatic encephalopathy, or hepatorenal syndrome and compensated cirrhosis defined by the absence of these features.

For antiplatelet strategy analyses, a 30‐day landmark approach was used among patients who survived beyond 30 days and had identifiable antiplatelet therapy. Monotherapy was defined as aspirin or a single P2Y12 inhibitor, while dual therapy consisted of aspirin plus a P2Y12 inhibitor at any time between 30 days and 1 year after PCI. Within the monotherapy cohort, aspirin and clopidogrel were compared, excluding patients who switched agents during follow‐up.

### 2.4 Statistical Methods

Statistical analyses were performed using the analytic functionality within the TriNetX platform. One‐to‐one propensity score matching was conducted using nearest‐neighbour matching with a calliper of 0.1 times the pooled standard deviation. After matching, categorical variables were compared using chi‐square tests and continuous variables using independent‐samples *t*‐test. Binary outcomes are reported as relative risks with 95% confidence intervals (CIs), and two‐sided *p* values less than 0.05 were considered statistically significant. Cox proportional hazards regression was used to evaluate clinical factors associated with 1‐year all‐cause mortality after PCI in patients with cirrhosis, with results reported as hazard ratios (HRs) and 95% CIs.

## 3 Results

### 3.1 Study Population and Baseline Characteristics

Among adults undergoing percutaneous coronary intervention (PCI) in the US Collaborative Network within TriNetX, 3351 patients with cirrhosis were identified (mean age 66.3 ± SD 9.2 years; 67.9% male) and compared with 324,274 patients without cirrhosis (mean age 66.1 ± SD 11.9 years; 68.4% male) (Figure 1, Table 1). Before matching, patients with cirrhosis had a higher prevalence of cardiovascular and metabolic comorbidities and greater use of anticoagulants and other cardiovascular medications. After 1:1 propensity score matching, 3351 patients with cirrhosis were well balanced with 3351 non‐cirrhosis controls across demographics and comorbidities (Table 1).

**FIGURE 1:**
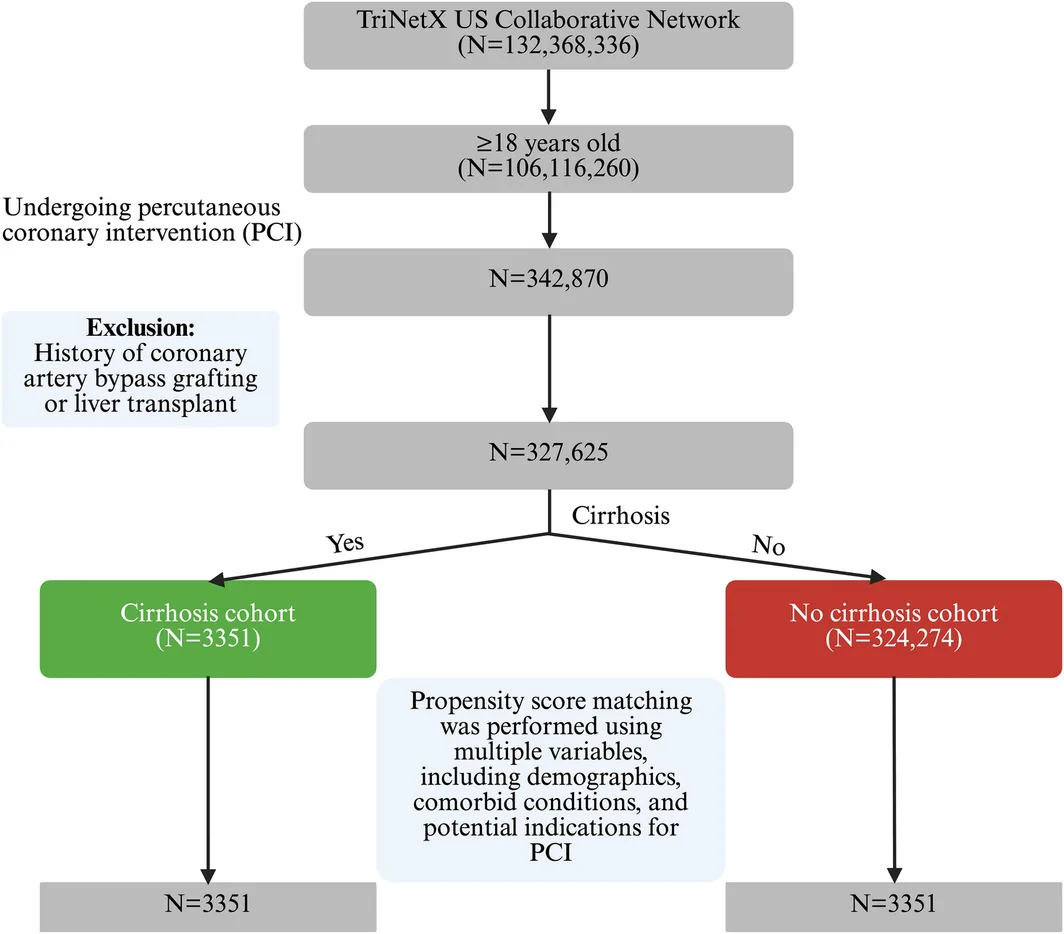
Cohort construction of patients with cirrhosis and non‐cirrhosis controls undergoing percutaneous coronary intervention (PCI) using the US Collaborative Network within TriNetX.

**TABLE 1:** Baseline characteristics of patients with and without cirrhosis undergoing percutaneous coronary intervention, before and after 1:1 propensity score matching.

	Characteristic	Cirrhosis cohort *N* (%)	Controls *N* (%)	*p*
Demographics	Age at testing	Mean 66.3 ± SD 9.2 (*N* = 3351)	Mean 66.1 ± SD 11.9 (*N* = 324,274)	0.23
		Mean 66.3 ± SD 9.2 (*N* = 3351)	Mean 66.3 ± SD 11.2 (*N* = 3351)	0.91
	Male	2275 (67.89)	221,983 (68.45)	0.48
		2275 (67.89)	2234 (66.67)	0.29
	White	2632 (78.54)	258,577 (79.74)	0.09
		2632 (78.54)	2616 (78.07)	0.64
	African American	440 (13.13)	33,294 (10.27)	**< 0.0001**
		440 (13.13)	459 (13.70)	0.50
	Asian	81 (2.42)	10,349 (3.19)	**0.01**
		81 (2.42)	78 (2.33)	0.81
	Unknown race	79 (2.36)	11,153 (3.44)	**0.0006**
		79 (2.36)	77 (2.30)	0.87
	Other race	77 (2.30)	7259 (2.24)	0.82
		77 (2.30)	76 (2.27)	0.93
Body mass index		Mean 30.1 ± SD 6.8 (*N* = 2787)	Mean 30.0 ± SD 6.4 (*N* = 242,501)	0.54
		Mean 30.1 ± SD 6.8 (*N* = 2787)	Mean 31.0 ± SD 7.0 (*N* = 2779)	**< 0.0001**
Haemoglobin		Mean 12.2 ± SD 2.4 (*N* = 3328)	Mean 13.3 ± SD 2.2 (*N* = 3328)	**< 0.0001**
		Mean 12.2 ± SD 2.4 (*N* = 3328)	Mean 12.7 ± SD 2.3 (*N* = 3328)	**< 0.0001**
Medications	Antilipemic agents	3042 (90.78)	282,297 (87.05)	**< 0.0001**
		3042 (90.78)	3040 (90.72)	0.93
	Apixaban	469 (14.00)	23,987 (7.40)	**< 0.0001**
		469 (14.00)	453 (13.52)	0.57
	Beta blockers	3022 (90.18)	245,345 (75.66)	**< 0.0001**
		3022 (90.18)	3036 (90.60)	0.56
	Dabigatran	33 (0.98)	1826 (0.56)	0.**001**
		33 (0.98)	23 (0.69)	0.18
	Enoxaparin	1589 (47.42)	8554 (26.38)	**< 0.0001**
		1589 (47.42)	1617 (48.25)	0.49
	Heparin	3193 (95.28)	293,718 (90.58)	**< 0.0001**
		3193 (95.28)	3182 (94.96)	0.53
	NSIADs	2049 (61.15)	117,940 (36.37)	**< 0.0001**
		2049 (61.15)	2119 (63.23)	0.08
	Proton pump inhibitor	2624 (78.30)	139,354 (42.97)	**< 0.0001**
		2624 (78.30)	2676 (79.86)	0.11
	Rivaroxaban	175 (5.22)	10,632 (3.28)	**< 0.0001**
		175 (5.22)	150 (4.48)	0.15
	Warfarin	372 (11.10)	18,109 (5.58)	**< 0.0001**
		372 (11.10)	344 (10.27)	0.27
Cardiovascular diseases	Acute MI	1889 (56.37)	190,643 (58.79)	**0.005**
		1889 (56.37)	1844 (55.03)	0.27
	Atrial fibrillation and flutter	931 (27.78)	60,942 (18.79)	**< 0.0001**
		931 (27.78)	900 (26.86)	0.40
	Cerebrovascular disease	1128 (33.66)	58,338 (17.99)	**< 0.0001**
		1128 (33.66)	1106 (33.00)	0.57
	Chronic IHD	3258 (97.22)	303,242 (93.51)	**< 0.0001**
		3258 (97.22)	3273 (97.67)	0.24
	Heart failure	1828 (54.55)	109,525 (33.77)	**< 0.0001**
		1828 (54.55)	1827 (54.52)	0.98
	Hypertension	3092 (92.27)	256,595 (79.13)	**< 0.0001**
		3092 (92.27)	3123 (93.20)	0.15
	NSTEMI	1425 (42.52)	117,663 (36.28)	**< 0.0001**
		1425 (42.52)	1399 (41.75)	0.52
	Other acute IHD	523 (15.61)	31,157 (9.61)	**< 0.0001**
		523 (15.61)	509 (15.19)	0.64
	Pulmonary embolism	159 (4.74)	7319 (2.26)	**< 0.0001**
		159 (4.74)	152 (4.54)	0.68
	STEMI of anterior wall	260 (7.76)	35,566 (10.97)	**< 0.0001**
		260 (7.76)	269 (8.03)	0.68
	STEMI of inferior wall	313 (9.34)	41,337 (12.75)	**< 0.0001**
		313 (9.34)	311 (9.28)	0.93
	STEMI of other sites	113 (3.37)	13,738 (4.24)	**0.01**
		113 (3.37)	97 (2.89)	0.26
	STEMI of unspecified site	581 (17.34)	68,562 (21.14)	**< 0.0001**
		581 (17.34)	564 (16.83)	0.58
	Unstable angina	743 (22.17)	56,497 (17.42)	**< 0.0001**
		743 (22.17)	720 (21.49)	0.50
Metabolic diseases	Type 1 diabetes mellitus	432 (12.89)	14,630 (4.51)	**< 0.0001**
		432 (12.89)	431 (12.86)	0.97
	Type 2 diabetes mellitus	2302 (68.70)	134,485 (41.47)	**< 0.0001**
		2302 (68.70)	2309 (68.90)	0.85
	Hyperlipidemia	2929 (87.41)	260,167 (80.23)	**< 0.0001**
		2929 (87.41)	2933 (87.53)	0.88
	Hypothyroidism	758 (22.62)	44,224 (13.64)	**< 0.0001**
		758 (22.62)	715 (21.34)	0.20
	Overweight/obesity	1761 (52.55)	98,058 (30.24)	**< 0.0001**
		1761 (52.55)	1765 (52.67)	0.92
Pulmonary diseases	COPD	1103 (32.92)	49,587 (15.29)	**< 0.0001**
		1103 (32.92)	1092 (32.59)	0.77
	Dependence on respirator	83 (2.48)	3359 (1.04)	**< 0.0001**
		83 (2.48)	70 (2.09)	0.29
Renal diseases	Chronic kidney disease	1500 (44.76)	71,275 (21.98)	**< 0.0001**
		1500 (44.76)	1490 (44.46)	0.81
	Dependence on renal dialysis	294 (8.77)	9531 (2.94)	**< 0.0001**
		294 (8.77)	291 (8.68)	0.90
Substance use disorders	Alcohol use disorder	918 (27.39)	16,469 (5.08)	**< 0.0001**
		918 (27.39)	893 (26.65)	0.49
	Cannabis use disorder	266 (7.94)	8183 (2.52)	**< 0.0001**
		266 (7.94)	244 (7.28)	0.31
	Cocaine use disorder	183 (5.46)	4000 (1.23)	**< 0.0001**
		183 (5.46)	172 (5.13)	0.55
	Nicotine dependence	1379 (41.15)	88,804 (27.38)	**< 0.0001**
		1379 (41.15)	1422 (42.43)	0.29
	Opioid use disorder	275 (8.21)	5443 (1.68)	**< 0.0001**
		275 (8.21)	244 (7.28)	0.16

*Note:* For each attribute, upper and lower row represents pre‐ and post‐propensity match data respectively. Bolded *p* values indicate statistically significant results.

Abbreviations: COPD, chronic obstructive pulmonary disease; IHD, ischaemic heart disease; MI, myocardial infarction; NSAID, nonsteroidal anti‐inflammatory drugs; NSTEMI, non‐ST‐segment elevation myocardial infarction; SD, standard deviation; STEMI, ST‐segment elevation myocardial infarction.

### 3.2 Primary Analysis: Outcomes After PCI in Cirrhosis Versus Non‐Cirrhosis Controls

At 30 days after PCI, patients with cirrhosis had higher healthcare utilisation and bleeding‐related outcomes, including increased intensive care unit admission, gastrointestinal bleeding, blood transfusion, and emergency department visits compared with matched controls. Rates of vasopressor support, inotropic support, stroke, encephalopathy, sepsis, acute kidney injury, and 30‐day mortality did not differ significantly between cohorts (Table 2).

**TABLE 2:** Comparison of percutaneous coronary intervention outcomes in patients with and without cirrhosis after 1:1 propensity matching.

Clinical outcomes		Cirrhosis cohort *N* (%)	Control *N* (%)	RR^a^	95% confidence interval	*p*
Total number of patients		3351	3351			
**30 days post PCI outcomes**						
Cardiovascular/hemodynamic instability	Advanced circulatory support	14 (0.42)	25 (0.75)	0.57	(0.31, 1.09)	0.08
	Vasopressor support	390 (11.64)	355 (10.59)	1.10	(0.96, 1.26)	0.17
	Inotropic support	243 (7.25)	230 (6.86)	1.06	(0.89, 1.26)	0.54
	ICU admission	576 (17.19)	501 (14.95)	1.15	(1.03, 1.28)	**0.01**
Haemorrhagic/thrombotic complications	Gastrointestinal bleeding	184 (5.49)	92 (2.74)	2.00	(1.56, 2.56)	**< 0.0001**
	Blood transfusion	156 (4.65)	106 (3.16)	1.47	(1.15, 1.87)	0.**002**
	Intracranial bleeding	12 (0.36)	13 (0.39)	0.92	(0.42, 2.02)	0.92
	Retroperitoneal hematoma	≤ 10^b^	≤ 10^b^	—	—	—
	Deep venous thrombosis	21 (0.63)	23 (0.69)	0.91	(0.51, 1.65)	0.76
	Pulmonary embolism	25 (0.75)	38 (1.13)	0.66	(0.40, 1.09)	0.10
Neurological complications	Cerebral infarction	85 (2.54)	100 (2.98)	0.85	(0.64, 1.13)	0.27
	Encephalopathy	66 (1.97)	56 (1.67)	1.17	(0.83, 1.68)	0.36
Other organ failure	Sepsis	75 (2.24)	64 (1.91)	1.17	(0.84, 1.63)	0.35
	Acute kidney injury	399 (11.91)	351 (10.47)	1.14	(0.99, 1.30)	0.06
Emergency department visit		551 (16.44)	454 (13.55)	1.21	(1.08, 1.36)	0.**0009**
Deceased		120 (3.58)	118 (3.52)	1.02	(0.79, 1.30)	0.89
**1 year post PCI outcomes**						
Haemorrhagic complications	Gastrointestinal bleeding	541 (16.14)	272 (8.12)	1.99	(1.73, 2.28)	**< 0.0001**
	Blood transfusion	406 (12.12)	226 (6.74)	1.80	(1.54, 2.10)	**< 0.0001**
	Intracranial bleeding	47 (1.40)	34 (1.01)	1.38	(0.89, 2.14)	0.15
Cardiovascular/ischaemic complications	Stent restenosis	270 (8.06)	235 (7.01)	1.15	(0.97, 1.36)	0.10
	CABG	22 (0.66)	39 (1.16)	0.56	(0.33, 0.95)	**0.03**
	Cerebral infarction	201 (6.00)	236 (7.04)	0.85	(0.71, 1.02)	0.08
Deceased		468 (13.97)	349 (10.41)	1.34	(1.18, 1.53)	**< 0.0001**

*Note:* Bolded *p* values indicate statistically significant results.

Abbreviations: CABG, coronary artery bypass grafting; ICU, intensive care unit; PCI, percutaneous coronary intervention.

^a^ The relative risk is calculated as patients with versus without cirrhosis prior to PCI.

^b^ To protect patient privacy, TriNetX rounds all counts ≤ 10 up to 10; as a result, comparisons involving these values should be interpreted with caution.

At 1 year, cirrhosis was associated with significantly higher rates of gastrointestinal bleeding (16.14% vs. 8.12%; RR 1.99), blood transfusion (12.12% vs. 6.74%; RR 1.80), and all‐cause mortality (13.97% vs. 10.41%; RR 1.34) (all *p* < 0.0001). Stent restenosis was numerically higher but not statistically different, while coronary artery bypass grafting was less frequent in patients with cirrhosis (Table 2).

In multivariable Cox analysis among patients with cirrhosis undergoing PCI, older age was associated with higher 1‐year mortality (HR 1.03, *p* < 0.0001). Among decompensation events, ascites (HR 1.31, *p* = 0.0045) and variceal bleeding (HR 1.25, *p* = 0.0306) were independently associated with mortality, while hepatorenal syndrome (HR 1.54, *p* = 0.0515), hepatic encephalopathy (HR 1.02, *p* = 0.8659), and spontaneous bacterial peritonitis (HR 1.11, *p* = 0.5806) showed positive but nonsignificant associations. PCI performed for acute coronary syndrome was associated with higher mortality, including NSTEMI (HR 1.25, *p* = 0.0283) and STEMI (HR 1.43, *p* < 0.0001). Higher creatinine, INR, and total bilirubin were associated with increased mortality, whereas higher albumin and sodium were protective (Figure 2).

**FIGURE 2:**
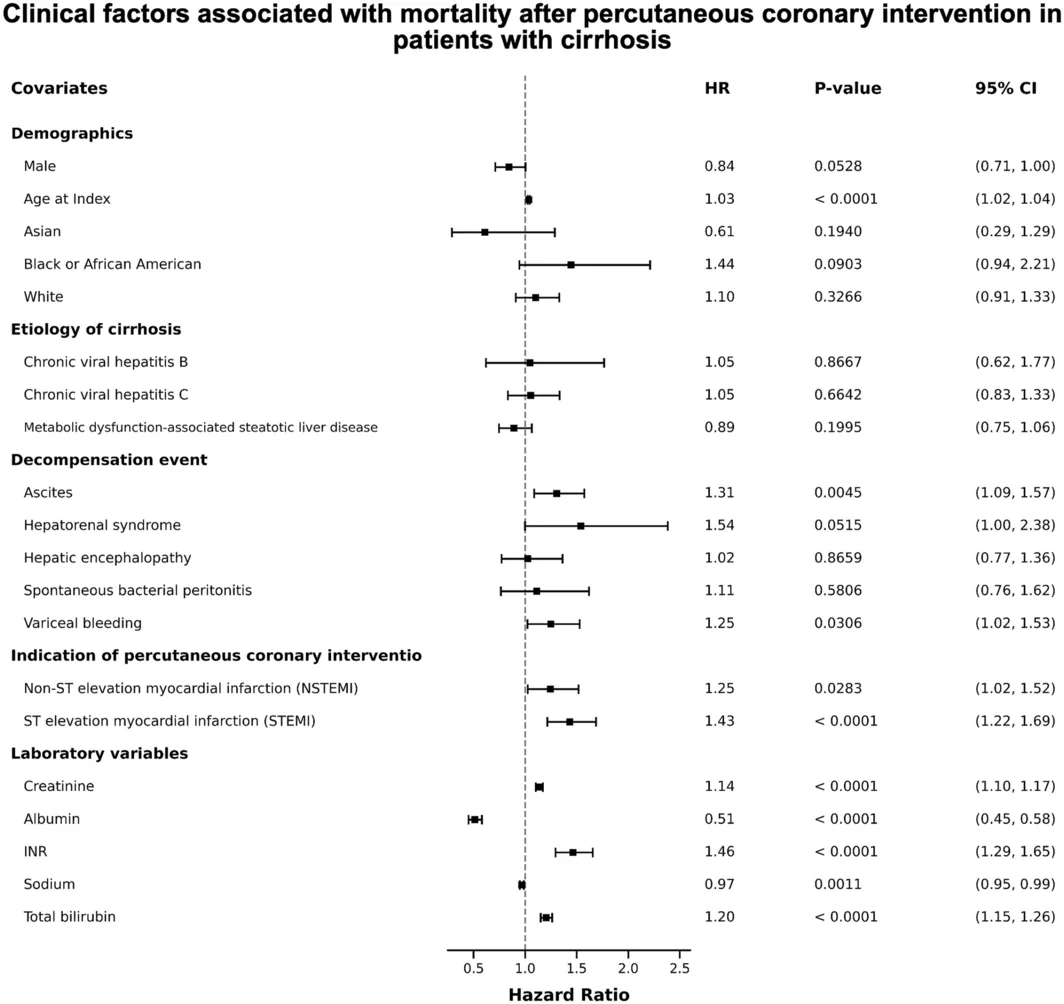
Clinical factors associated with 1‐year all‐cause mortality after percutaneous coronary intervention among patients with cirrhosis.

### 3.3 Subgroup Analyses Within the Cirrhosis Cohort

Subgroup analyses were performed among cirrhosis patients undergoing PCI (Figure 3), with outcomes assessed at 1 year (Table 3).

**FIGURE 3:**
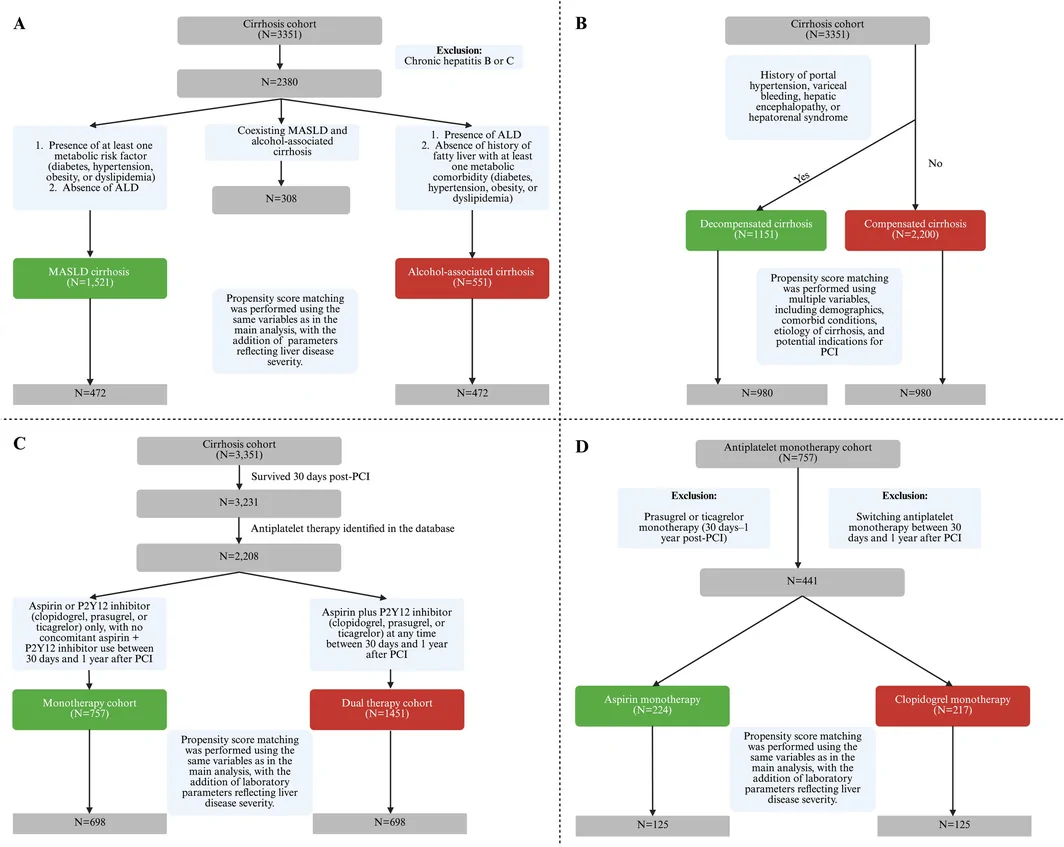
Cohort construction for subgroup analyses among cirrhosis patients undergoing percutaneous coronary intervention (PCI) using the US Collaborative Network within TriNetX: (A) cirrhosis aetiology, (B) compensated versus decompensated cirrhosis, (C) antiplatelet monotherapy versus dual antiplatelet therapy, and (D) aspirin versus clopidogrel monotherapy.

**TABLE 3:** Post–propensity score–matched clinical outcomes after percutaneous coronary intervention among patients with cirrhosis, stratified by cirrhosis aetiology, severity, and antiplatelet therapy strategy.

Clinical outcomes		Cohort 1 *N* (%)	Cohort 2 *N* (%)	RR^a^	95% confidence interval	*p*
		Alcohol‐associated cirrhosis	MASLD cirrhosis			
Total number of patients		472	472			
Haemorrhagic complications	GI bleeding	94 (19.92)	71 (15.04)	1.34	(1.01, 1.78)	**0.04**
	Blood transfusion	77 (16.31)	52 (11.02)	1.48	(1.06, 2.07)	**0.02**
Cardiovascular/ischaemic complications	Stent restenosis	33 (7.00)	43 (9.11)	0.77	(0.50, 1.19)	0.23
	Cerebral infarction	26 (5.51)	28 (5.93)	0.93	(0.55, 1.56)	0.78
Deceased		81 (17.14)	73 (15.38)	1.11	(0.83, 1.50)	0.47

		Decompensated cirrhosis	Compensated cirrhosis			
Total number of patients		980	980			
Haemorrhagic complications	GI bleeding	233 (23.78)	109 (11.12)	2.14	(1.73, 2.64)	**< 0.0001**
	Blood transfusion	199 (17.91)	101 (9.09)	1.97	(1.57, 2.47)	**< 0.0001**
Cardiovascular/ischaemic complications	Stent restenosis	93 (9.49)	66 (6.73)	1.41	(1.04, 1.91)	**0.03**
	Cerebral infarction	49 (5.00)	53 (5.41)	0.92	(0.63, 1.35)	0.68
Deceased		188 (19.18)	110 (11.22)	1.71	(1.37, 2.12)	**< 0.0001**

		Antiplatelet monotherapy	Antiplatelet dual therapy			
Total number of patients		698	698			
Haemorrhagic complications	GI bleeding	72 (10.31)	145 (20.77)	0.50	(0.38, 0.65)	**< 0.0001**
	Blood transfusion	57 (8.17)	103 (14.76)	0.55	(0.41, 0.75)	**< 0.0001**
Cardiovascular/ischaemic complications	Stent restenosis	62 (8.88)	49 (7.02)	1.13	(0.93, 1.32)	0.20
	Cerebral infarction	42 (6.02)	46 (6.59)	0.95	(0.75, 1.17)	0.66
Deceased		66 (9.46)	90 (12.89)	0.73	(0.54, 0.99)	**0.04**

		Aspirin monotherapy	Clopidogrel monotherapy			
Total number of patients		125	125			
Haemorrhagic complications	GI bleeding	21 (17.50)	14 (11.20)	1.56	(0.80, 2.60)	0.23
	Blood transfusion	18 (14.40)	12 (9.60)	1.50	(0.75, 2.98)	0.24
Cardiovascular/ischaemic complications	Stent restenosis	≤ 10^b^	≤ 10^b^	—	—	—
	Cerebral infarction	≤ 10^b^	≤ 10^b^	—	—	—
Deceased		21 (16.80)	13 (10.40)	1.61	(0.85, 3.08)	0.14

*Note:* Bolded *p* values indicate statistically significant results.

Abbreviations: CABG, coronary artery bypass grafting; GI, gastrointestinal; ICU, intensive care unit; MASLD, metabolic dysfunction‐associated steatotic liver disease; PCI, percutaneous coronary intervention.

^a^ The relative risk is calculated as patients with versus without cirrhosis prior to PCI.

^b^ To protect patient privacy, TriNetX rounds all counts ≤ 10 up to 10; as a result, comparisons involving these values should be interpreted with caution.

### 3.4 Aetiology‐Based Comparison (Alcohol‐Associated Vs. MASLD‐Related Cirrhosis)

Before propensity score matching, patients with alcohol‐associated cirrhosis had a higher prevalence of portal hypertension and oesophageal varices compared with those with MASLD‐related cirrhosis. These differences, along with other baseline demographic and clinical characteristics, were balanced after propensity score matching (Table S2).

After propensity matching (472 per group), alcohol‐associated cirrhosis was associated with higher 1‐year GI bleeding (19.92% vs. 15.04%; RR 1.34; *p* = 0.04) and blood transfusion (16.31% vs. 11.02%; RR 1.48; *p* = 0.02) compared to MASLD‐related cirrhosis. One‐year stent restenosis, cerebral infarction, and mortality were similar between groups (Table 3).

### 3.5 Cirrhosis Severity‐Based Comparison (Decompensated Vs. Compensated)

Before propensity score matching, patients with decompensated cirrhosis were younger and more often had heart failure, chronic kidney disease, dialysis dependence, alcohol‐associated cirrhosis, whereas compensated cirrhosis was more commonly associated with chronic viral hepatitis; these characteristics were well balanced after matching (Table S3).

After propensity score matching (980 per group), decompensated cirrhosis was associated with higher 1‐year gastrointestinal bleeding (23.78% vs. 11.12%; RR 2.14), blood transfusion (17.91% vs. 9.09%; RR 1.97), and mortality (19.18% vs. 11.22%; RR 1.71) compared with compensated cirrhosis (all *p* < 0.0001), as well as higher stent restenosis (9.49% vs. 6.73%; RR 1.41; *p* = 0.03) (Table 3).

### 3.6 Antiplatelet Strategy‐Based Comparison (Monotherapy Vs. Dual Antiplatelet Therapy After 30 Days)

Before propensity score matching, patients later managed with dual antiplatelet therapy more often presented with acute coronary syndromes and cardiovascular comorbidities, whereas those managed with antiplatelet monotherapy more frequently had atrial fibrillation and oral anticoagulant use. After matching, baseline characteristics were well balanced between groups (Table S4).

In the 30‐day landmark analysis (698 per group), antiplatelet monotherapy was associated with lower rates of gastrointestinal bleeding (10.31% vs. 20.77%; RR 0.50) and blood transfusion (8.17% vs. 14.76%; RR 0.55) compared with dual therapy (both *p* ≤ 0.0001). One‐year mortality was also lower with monotherapy (9.46% vs. 12.89%; RR 0.73; *p* = 0.04), while rates of stent restenosis and cerebral infarction were similar (Table 3).

### 3.7 Aspirin Versus Clopidogrel Monotherapy‐Based Comparison

Before propensity score matching, patients later managed with clopidogrel monotherapy were older and more frequently had atrial fibrillation and heart failure, whereas aspirin monotherapy was more common among patients presenting with ST‐segment elevation myocardial infarction at PCI; these differences were well balanced after matching (Table S5).

Among matched monotherapy recipients, gastrointestinal bleeding, transfusion, and mortality were numerically higher with aspirin compared with clopidogrel, although these differences did not reach statistical significance (Table 3).

### 3.8 Kaplan–Meier Curves for Gastrointestinal Bleeding and Mortality After PCI

Kaplan–Meier curves depicting the 1‐year risk of gastrointestinal bleeding and all‐cause mortality after PCI in the primary cohort and subgroup analyses are shown in Figure 4 and the Figure S1, respectively.

**FIGURE 4:**
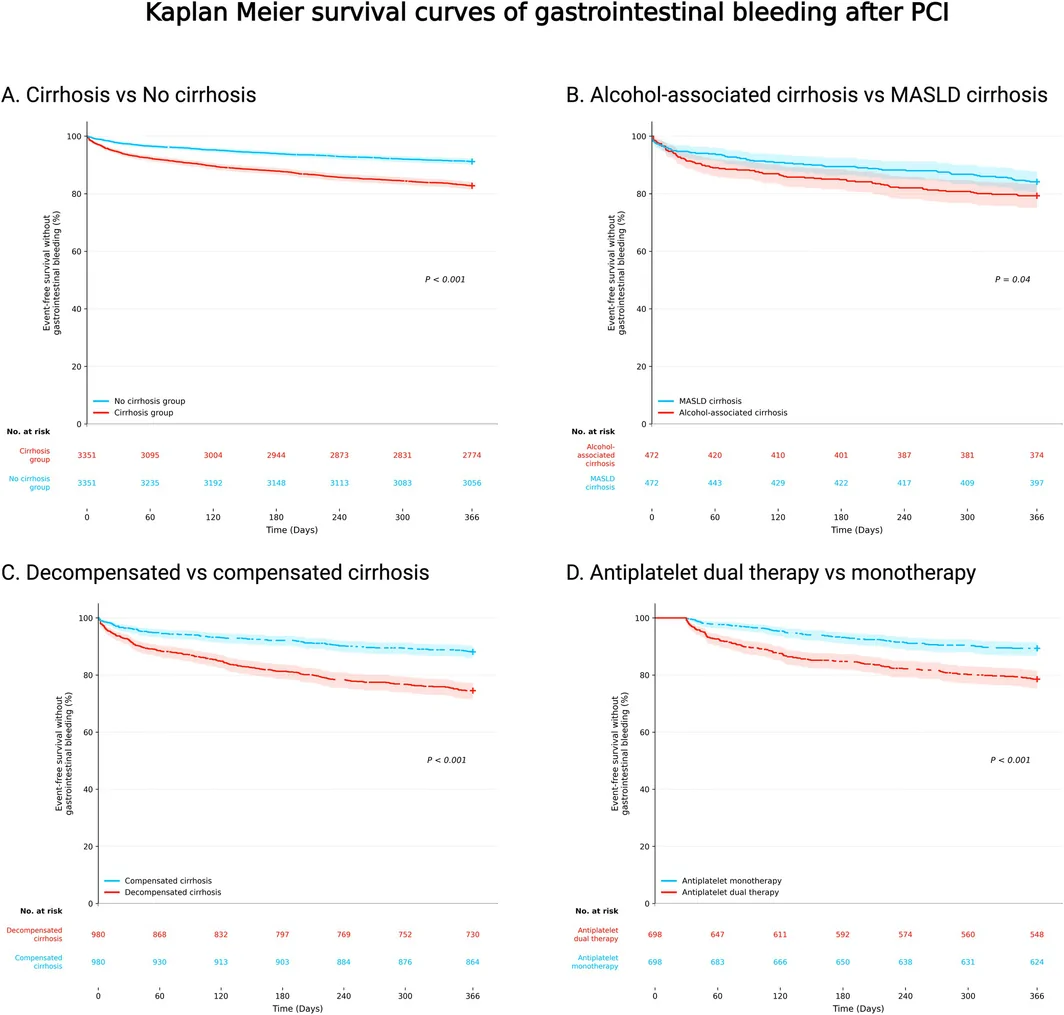
Kaplan–Meier curves depicting the 1‐year risk of gastrointestinal (GI) bleeding among patients undergoing percutaneous coronary intervention (PCI) in the primary cohort and subgroup analyses: (A) cirrhosis versus non‐cirrhosis patients, (B) cirrhosis aetiology, (C) decompensated versus compensated cirrhosis, and (D) dual antiplatelet therapy versus monotherapy.

## 4 Discussion

In this propensity‐matched study, patients with cirrhosis undergoing PCI had a twofold higher risk of gastrointestinal bleeding and a 34% higher risk of mortality within 1 year. Subgroup analyses revealed important risk stratification patterns, with alcohol‐associated cirrhosis patients demonstrating a higher risk of gastrointestinal bleeding and a nonsignificant trend toward higher 1‐year mortality compared with those with MASLD‐related cirrhosis. In addition, patients with decompensated cirrhosis had more than a twofold higher risk of gastrointestinal bleeding and a 71% higher risk of mortality within 1 year compared with those with compensated cirrhosis. Furthermore, antiplatelet monotherapy after 30 days post‐PCI was associated with significantly lower risks of bleeding and 1‐year mortality compared with dual antiplatelet therapy, without an increased rate of thrombotic events. These findings have important implications for risk stratification, shared decision‐making, and antiplatelet management in patients with cirrhosis undergoing PCI.

The increased risks of gastrointestinal bleeding and mortality observed in our cirrhotic cohort are consistent with findings from prior meta‐analyses demonstrating higher rates of gastrointestinal bleeding and in‐hospital mortality following PCI in patients with cirrhosis [24]. The mechanisms underlying this elevated bleeding risk are multifactorial and include portal hypertension, coagulopathy, thrombocytopenia, and platelet dysfunction. Among these, portal hypertension is a key contributor, promoting bleeding through several pathways such as the development of oesophageal and gastric varices, portal hypertensive gastropathy, and ectatic vessels throughout the gastrointestinal tract [28]. According to the Academic Research Consortium for High Bleeding Risk (ARC‐HBR), cirrhosis patients are classified as Bleeding Academic Research Consortium (BARC) type 3 or 5 bleeding risk. BARC type 3 bleeding represents major bleeding requiring transfusion or medical intervention, whereas type 5 bleeding represents fatal bleeding [29]. The additional exposure to antiplatelet therapy in this already high‐risk population likely explains the nearly twofold increase in gastrointestinal bleeding observed at both 30 days and 1 year in our study.

The increased mortality observed in cirrhosis patients post‐PCI likely represents a complex interaction of bleeding complications, underlying cardiac dysfunction, and reduced physiologic reserve. Cirrhotic cardiomyopathy, present in up to 50% of patients with advanced liver disease, is characterised by QT interval prolongation, impaired contractile response to stress, and beta‐adrenergic receptor desensitisation [28, 30, 31, 32]. In addition, acute kidney injury occurs in approximately 28.4% of PCI procedures among patients with cirrhosis compared with 10.9% in the general STEMI‐PCI population, with dialysis required in 4.9% versus 0.8%, respectively [21, 33]. Importantly, cardiovascular disease has emerged as the second leading cause of death among patients with cirrhosis and the leading cause of mortality in those with MASLD‐related cirrhosis [34, 35].

In our aetiology‐driven subgroup analysis, patients with alcohol‐associated cirrhosis had a higher risk of gastrointestinal bleeding and blood transfusion compared with those with MASLD‐related cirrhosis, along with a nonsignificant trend toward higher mortality. These findings may reflect differences in portal hypertension severity, ongoing alcohol exposure, or other unmeasured clinical factors [36, 37]. Notably, recent meta‐analyses demonstrate that alcohol abstinence reduces the risk of portal hypertensive complications and confers a clear survival benefit in patients with alcohol‐associated cirrhosis, underscoring the modifiable nature of this risk and the importance of counselling regarding alcohol abstinence in patients with alcohol‐associated cirrhosis undergoing PCI [38, 39].

Decompensated cirrhosis was associated with significantly higher risks of gastrointestinal bleeding, transfusion, all‐cause mortality, and stent restenosis, highlighting the uniquely elevated procedural risk in this population. This relationship was further supported by our Cox analysis, in which ascites and variceal bleeding were independently associated with mortality after PCI, while hepatorenal syndrome, hepatic encephalopathy, and spontaneous bacterial peritonitis showed positive but nonsignificant associations. The marked differences in outcomes between compensated and decompensated cirrhosis underscore liver disease severity as an important determinant in PCI decision‐making and support viewing cirrhosis as a continuous rather than categorical condition. Incorporation of Child–Pugh, Vocal‐Penn, and MELD scores into pre‐procedural assessment, along with multidisciplinary discussion, modulation of portal hypertension, and consideration of noninvasive alternatives, may help guide patient selection.

In addition to liver disease severity, the indication for PCI should also be considered, as PCI for acute coronary syndromes, including NSTEMI and STEMI, was independently associated with higher mortality.

The most clinically actionable findings from our study relate to antiplatelet management. In a 30‐day landmark analysis, antiplatelet monotherapy (aspirin or clopidogrel) was associated with a 50% lower risk of gastrointestinal bleeding, a 45% lower risk of transfusion, and a 27% lower risk of mortality compared with dual antiplatelet therapy, without higher rates of stent restenosis or cerebral infarction at 1 year. These findings align with prior evidence and current American College of Cardiology/American Heart Association and European Society of Cardiology guidelines recommending 1–3 months of dual antiplatelet therapy in high bleeding risk patients followed by P2Y12 inhibitor monotherapy [40, 41]. Although no statistically significant differences were observed between aspirin and clopidogrel monotherapy, likely due to limited sample size, aspirin monotherapy showed a consistent numerical trend toward higher risks of gastrointestinal bleeding, transfusion, and mortality, consistent with prior literature suggesting clopidogrel may be preferable in high bleeding risk patients [42, 43, 44]. Additional strategies to mitigate bleeding risk include proton pump inhibitor co‐therapy and preferential radial access during PCI [45, 46, 47, 48].

This study has several important strengths, including a large real‐world cohort drawn from 72 healthcare institutions across the United States, the use of propensity score matching to reduce confounding, evaluation of clinically relevant outcomes, and prespecified subgroup analyses by cirrhosis aetiology, disease severity, and antiplatelet strategy. However, several limitations should be considered. As a retrospective observational study, residual confounding from unmeasured or imbalanced variables may persist despite propensity matching. Although patients with prior liver transplantation were excluded, we could not reliably censor patients who underwent liver transplantation after PCI during follow‐up. Therefore, 1‐year mortality was analysed as all‐cause mortality without censoring for interval transplantation. Cause‐specific mortality was also unavailable, limiting our ability to distinguish cardiac, liver‐related, and other causes of death. Key procedural details, including stent type and vascular access site, were unavailable, and cirrhosis‐specific measures such as hepatic venous pressure gradient and the grade of varices could not be assessed, limiting more granular risk stratification. We attempted to account for liver disease severity using Model for End‐Stage Liver Disease–related laboratory parameters when relevant; however, direct matching on Model for End‐Stage Liver Disease score is not feasible within the TriNetX platform. The landmark analysis of antiplatelet strategies was restricted to patients identifiable as receiving specific regimens, which may introduce selection bias, and comparisons between aspirin and clopidogrel monotherapy were likely underpowered. In addition, administrative data did not permit assessment of medication adherence, dose adjustments, or clinical rationale for treatment selection. Finally, as with other database studies, our findings may be subject to surveillance and immortal time bias.

In conclusion, this large propensity‐matched cohort study demonstrates that cirrhosis is associated with substantially elevated risks of bleeding and mortality following PCI, with outcomes strongly influenced by disease severity and aetiology. Decompensated cirrhosis confers a particularly poor prognosis, underscoring the importance of careful risk stratification in clinical decision‐making. Our findings support abbreviated dual antiplatelet therapy strategies in patients with cirrhosis, with antiplatelet monotherapy after 1 month associated with significant reductions in bleeding and mortality without an increased risk of thrombotic events. As the prevalence of cirrhosis continues to rise and cardiovascular disease emerges as a leading cause of death in this population, these findings provide evidence‐based guidance for managing the complex intersection of coronary artery disease and advanced liver disease. Future prospective randomised trials are needed to definitively establish optimal antiplatelet regimens, the duration of dual therapy, and the comparative effectiveness of medical versus invasive management strategies in patients with cirrhosis and coronary artery disease.

## Author Contributions

**Manas Gunani:** conceptualization, software, formal analysis. **Anand V. Kulkarni:** conceptualization, writing – review and editing. **Kaveh Hajifathalian:** conceptualization, writing – review and editing. **Kajol Shah:** conceptualization, writing – review and editing. **Dhir Gala:** conceptualization, writing – original draft, methodology, visualization. **Daniel Q. Huang:** conceptualization, writing – review and editing. **Nikki Duong:** conceptualization, writing – review and editing, supervision. **Pojsakorn Danpanichkul:** conceptualization, writing – review and editing. **Paul Gaglio:** conceptualization, writing – review and editing. **Sameer Rao:** conceptualization, methodology, writing – original draft, visualization, formal analysis. **Karn Wijarnpreecha:** conceptualization, writing – review and editing. **Vivek Lingiah:** conceptualization, writing – review and editing. **Christine Gerula:** conceptualization, writing – review and editing, supervision. **Ahmed Al‐Khazraji:** conceptualization, writing – review and editing.

## Funding

The authors have nothing to report.

## Disclosure

*Guarantor of the article*: Sameer Rao.

## Ethics Statement

The authors have nothing to report.

## Conflicts of Interest

An abstract based on this study was presented as a poster at Digestive Disease Week 2026 in Chicago, IL, USA. Dr. Huang, a co‐author of this manuscript, serves as an Associate Editor for Alimentary Pharmacology & Therapeutics. The authors have no other conflicts of interest to disclose.

## Supporting information


**Table S1:** Attributes and corresponding administrative codes used in the study.
**Table S2:** Baseline characteristics of patients with alcohol‐associated cirrhosis and metabolic dysfunction‐associated steatotic liver disease (MASLD) cirrhosis undergoing percutaneous coronary intervention, before and after 1:1 propensity score matching.
**Table S3:** Baseline characteristics of cirrhosis patients undergoing percutaneous coronary intervention (PCI), stratified by severity of cirrhosis, before and after propensity score matching.
**Table S4:** Baseline characteristics of cirrhosis patients undergoing percutaneous coronary intervention (PCI), stratified by antiplatelet monotherapy versus dual therapy after 1 month of PCI, before and after propensity score matching.
**Table S5:** Baseline characteristics of cirrhosis patients undergoing percutaneous coronary intervention (PCI), stratified by aspirin versus clopidogrel monotherapy after 1 month of PCI, before and after propensity score matching.

**Figure S1:** Kaplan–Meier curves depicting the 1‐year risk of mortality among patients undergoing percutaneous coronary intervention (PCI) in the primary cohort and subgroup analyses: (A) cirrhosis versus non‐cirrhosis patients, (B) cirrhosis aetiology, (C) compensated versus decompensated cirrhosis, (D) antiplatelet monotherapy versus dual antiplatelet therapy, and (E) aspirin versus clopidogrel monotherapy.

## Data Availability

Data, analytic methods, and study materials will not be made available to other researchers. Analyses were performed within the TriNetX platform, which provides only de‐identified, aggregate‐level data that cannot be exported or shared outside the platform.
