# Supplementary material for: Clinical Outcomes After Percutaneous Coronary Intervention in Patients With Cirrhosis: A Multicentre National Study
Source: Aliment Pharmacol Ther. 2026 Aug 5;64(9):1219–33. doi: 10.1111/apt.70896 (PMC13651923; doi:10.1111/apt.70896)
Supplement: Supplementary file 1 [file APT-64-1219-s001.docx]

**Supplementary Data**

**Supplementary Table 1 -** Attributes and corresponding administrative codes used in the study.

| Attribute | ICD-10/CPT/LOINC/RxNorm |
| --- | --- |
|  |  |
| Acute myocardial infarction | I21 |
| Acute myocardial infarction, unspecified | I21.9 |
| Alcohol use disorder | F10 |
| Alcoholic cirrhosis | K70.3 |
| Alcoholic liver disease | K70 |
| Antilipidemic agents | CV350 |
| Apixaban | 1364430 |
| Ascites | R18 |
| Aspirin | 1191 |
| Atrial fibrillation | I48 |
| Beta blocker | CV100 |
| Cannabis use disorder | F12 |
| Cardiac stent restenosis | T82.85 |
| Cerebrovascular disease | I60-I69 |
| Chronic ischemic disease | I25 |
| Chronic kidney disease | N18 |
| Chronic obstructive pulmonary disease | J44.9 |
| Chronic viral hepatitis B | B18.0, B18.1 |
| Chronic viral hepatitis C | B18.2 |
| Cirrhosis | K74.6, K70.3, K74.69 |
| Clopidogrel | 32968 |
| Cocaine use disorder | F14 |
| Coronary artery bypass grafting | 33513, 33514, 33533, 33521, 33512, 1006216, 33516, 33510, 33513, 33522, 33518, 33512, 33535, 33534, 33523, 33511, 33530, 33519, 33536, 33517 |
| Creatinine | 9024 |
| Dabigatran | 1037042 |
| Dependence on renal dialysis | Z99.2 |
| Dependence on respirator | Z99.1 |
| Diabetes | E08-E13 |
| Disorders of lipid metabolism | E78 |
| Enoxaparin | 67108 |
| Esophageal varices | I85 |
| Essential hypertension | I10 |
| Fatty liver | K76.0 |
| Gastrointestinal bleeding | K92.1, K92.2, K92.0, I85.01 |
| Heart failure | I50 |
| Hemoglobin | 9014 |
| Heparin | 5224 |
| Hepatic encephalopathy | K72.90, K72.91 |
| Hepatorenal syndrome | K76.7 |
| Hypothyroidism | E03.9 |
| International normalized ratio | 9032 |
| MASH | K75.81 |
| Milrinone | 52769 |
| Nicotine use disorder | F17 |
| Non-ST elevation myocardial infarction | I21.4 |
| Nonsteroidal Anti-Inflammatory Drugs | MS102 |
| Obesity | E66.9 |
| Opioid use disorder | F11 |
| Other acute ischemic heart diseases | I24 |
| Overweight | E66 |
| Percutaneous coronary intervention | 92924, 92925, 92928, 92929, 92933, 92934, 92941, 92943, 92944 |
| Portal hypertension | K76.6 |
| Prasugrel | 613391 |
| Proton pump inhibitor | A02BC |
| Pulmonary embolism | I26 |
| Rivaroxaban | 1114195 |
| Sodium | 9029 |
| STEMI of anterior wall | I21.0 |
| STEMI of inferior wall | I21.1 |
| STEMI of other sites | I21.2 |
| STEMI of unspecified site | I21.3 |
| Ticagrelor | 1116632 |
| Total bilirubin | 9050 |
| Type 1 diabetes | E10 |
| Type 2 diabetes | E11 |
| Unstable angina | I20.0 |
| Variceal bleeding | I85.01, I85.11 |
| Warfarin | 11289 |
|  |  |
| Abbreviations: CPT - current procedural terminology, ICD-10 - international classification of diseases, 10th revision, LOINC - logical observation identifiers names and codes, MASH - metabolic dysfunction-associated steatohepatitis, STEMI – ST-segment elevation myocardial infarction. | |
|  | |

**Supplementary Table 2 –** Baseline characteristics of patients with alcohol-associated cirrhosis and metabolic dysfunction-associated steatotic liver disease (MASLD) cirrhosis undergoing percutaneous coronary intervention, before and after 1:1 propensity score matching.

|  | **Characteristic** | **Alcohol-associated cirrhosis**  **N (%)** | **MASLD**  **cirrhosis**  **N (%)** | ***p*-value** |
| --- | --- | --- | --- | --- |
|  |  |  |  |  |
| **Demographics** | Age at testing | Mean 64.7 ± SD 8.6  (N=551) | Mean 68.1 ± SD 9.9  (N=1,521) | **< 0.0001** |
|  |  | Mean 65.3 ± SD 8.5  (N=472) | Mean 65.3 ± SD 10.0  (N=472) | 1 |
|  | Male | 456 (83.06) | 863 (56.74) | **< 0.0001** |
|  |  | 382 (80.93) | 401 (84.96) | 0.42 |
|  | White | 443 (80.69) | 1,281 (84.22) | 0.06 |
|  |  | 392 (83.05) | 401 (84.96) | 0.42 |
|  | African American | 65 (11.84) | 119 (7.82) | **0.005** |
|  |  | 44 (9.32) | 44 (9.32) | 1 |
|  | Asian | 10 (1.82) | 41 (2.70) | 0.26 |
|  |  | 10 (2.12) | 10 (2.12) | 1 |
|  | Unknown race | 12 (2.19) | 37 (2.43) | 0.74 |
|  |  | 11 (2.33) | 10 (2.12) | 0.82 |
|  | Other race | 17 (3.10) | 29 (1.91) | 0.10 |
|  |  | 13 (2.75) | 10 (2.12) | 0.53 |
| **Body mass index** | | Mean 28.3 ± SD 6.0  (N=448) | Mean 31.3 ± SD 7.0  (N=1,279) | **< 0.0001** |
|  |  | Mean 28.5 ± SD 6.0  (N=384) | Mean 31.2 ± SD 6.4  (N=396) | **< 0.0001** |
| **Laboratory parameters** | Creatinine | Mean 1.5 ± SD 1.8  (N=534) | Mean 1.6 ± SD 1.9  (N=1,506) | 0.34 |
|  |  | Mean 1.6 ± SD 1.9  (N=462) | Mean 1.6 ± SD 1.9  (N=465) | 0.73 |
|  | Hemoglobin | Mean 12.0 ± SD 2.6  (N=545) | Mean 12.1 ± SD 2.4  (N=1,510) | 0.23 |
|  |  | Mean 11.9 ± SD 2.4  (N=468) | Mean 12.6 ± SD 2.5  (N=470) | **< 0.0001** |
|  | International normalized ratio | Mean 1.2 ± SD 0.2  (N=521) | Mean 1.2 ± SD 0.3  (N=1,421) | **0.04** |
|  |  | Mean 1.2 ± SD 0.2  (N=445) | Mean 1.2 ± SD 0.4  (N=453) | 0.33 |
|  | Sodium | Mean 137.0 ± SD 4.0  (N=543) | Mean 138.0 ± SD 3.6  (N=1,515) | **< 0.0001** |
|  |  | Mean 136.0 ± SD 4.0  (N=468) | Mean 137.0 ± SD 3.5  (N=470) | **0.0002** |
|  | Total bilirubin | Mean 1.0 ± SD 1.1  (N=532) | Mean 0.9 ± SD 1.1  (N=1,468) | **0.002** |
|  |  | Mean 1.0 ± SD 1.1  (N=456) | Mean 0.9 ± SD 1.5  (N=459) | 0.23 |
| **Medications** | Apixaban | 74 (13.48) | 246 (16.17) | 0.13 |
|  |  | 71 (15.04) | 76 (16.10) | 0.65 |
|  | Beta blockers | 504 (91.80) | 1,368 (89.94) | 0.20 |
|  |  | 431 (91.31) | 434 (91.95) | 0.72 |
|  | Dabigatran | 10 (1.82) | 13 (0.85) | 0.06 |
|  |  | 10 (2.12) | 10 (2.12) | 1 |
|  | Enoxaparin | 276 (50.27) | 724 (47.60) | 0.28 |
|  |  | 233 (49.36) | 226 (47.88) | 0.65 |
|  | Heparin | 524 (95.45) | 1,451 (95.40) | 0.96 |
|  |  | 450 (95.34) | 455 (96.40) | 0.41 |
|  | NSAIDs | 329 (59.93) | 903 (59.37) | 0.82 |
|  |  | 274 (58.05) | 273 (57.84) | 0.95 |
|  | Proton pump inhibitor | 440 (79.85) | 1219 (78.95) | 0.68 |
|  |  | 373 (79.02) | 371 (78.60) | 0.87 |
|  | Rivaroxaban | 26 (4.74) | 92 (6.05) | 0.26 |
|  |  | 24 (5.08) | 22 (4.66) | 0.76 |
|  | Warfarin | 51 (9.29) | 191 (12.56) | **0.04** |
|  |  | 47 (9.96) | 49 (10.38) | 0.83 |
| **Cardiovascular**  **diseases** | Acute MI | 320 (58.29) | 829 (54.50) | 0.13 |
|  |  | 270 (57.20) | 263 (55.72) | 0.65 |
|  | Atrial fibrillation | 153 (27.87) | 475 (31.23) | 0.14 |
|  |  | 137 (29.02) | 145 (30.72) | 0.57 |
|  | Cerebrovascular diseases | 171 (31.15) | 557 (36.62) | **0.02** |
|  |  | 152 (32.20) | 149 (31.57) | 0.83 |
|  | Chronic IHD | 528 (96.17) | 1,483 (97.50) | 0.11 |
|  |  | 455 (96.40) | 452 (95.76) | 0.61 |
|  | Heart failure | 313 (57.01) | 868 (57.07) | 0.98 |
|  |  | 269 (56.99) | 271 (57.41) | 0.89 |
|  | NSTEMI | 233 (42.44) | 632 (41.55) | 0.72 |
|  |  | 199 (42.16) | 190 (40.25) | 0.55 |
|  | Other acute IHD | 77 (14.03) | 236 (15.52) | 0.40 |
|  |  | 71 (15.04) | 62 (13.14) | 0.40 |
|  | Pulmonary embolism | 27 (4.92) | 75 (4.93) | 0.99 |
|  |  | 22 (4.66) | 29 (6.14) | 0.31 |
|  | STEMI of anterior wall | 50 (9.11) | 96 (6.31) | 0.03 |
|  |  | 34 (7.20) | 37 (7.84) | 0.71 |
|  | STEMI of inferior wall | 61 (11.11) | 132 (8.68) | 0.09 |
|  |  | 49 (10.38) | 58 (12.29) | 0.36 |
|  | STEMI of other sites | 17 (3.10) | 49 (3.22) | 0.89 |
|  |  | 15 (3.18) | 19 (4.02) | 0.48 |
|  | STEMI of unspecified site | 108 (19.67) | 247 (16.24) | 0.07 |
|  |  | 86 (18.22) | 87 (18.43) | 0.93 |
|  | Unstable angina | 97 (17.67) | 380 (24.98) | **0.0005** |
|  |  | 92 (19.49) | 90 (19.07) | 0.87 |
| **Gastrointestinal and liver diseases** | Esophageal varices | 182 (33.15) | 281 (18.46) | **< 0.0001** |
|  |  | 176 (37.29) | 166 (35.17) | 0.52 |
|  | Portal hypertension | 233 (42.44) | 369 (24.26) | **< 0.0001** |
|  |  | 176 (37.29) | 166 (35.17) | 0.52 |
| **Pulmonary diseases** | COPD | 207 (37.70) | 465 (30.57) | **0.002** |
|  |  | 170 (36.02) | 166 (35.17) | 0.79 |
|  | Dependence on respirator | 14 (2.55) | 32 (2.10) | 0.54 |
|  |  | 12 (2.54) | 13 (2.75) | 0.84 |
| **Renal diseases** | Chronic kidney disease | 237 (43.17) | 722 (47.47) | 0.08 |
|  |  | 210 (44.49) | 211 (44.70) | 0.95 |
|  | Dependence on renal dialysis | 48 (8.74) | 136 (8.94) | 0.89 |
|  |  | 43 (9.11) | 43 (9.11) | 1 |
| **Substance use disorders** | Cannabis use disorder | 60 (10.93) | 55 (3.62) | **< 0.0001** |
|  |  | 39 (8.05) | 39 (8.05) | 1 |
|  | Cocaine use disorder | 45 (8.20) | 16 (1.05) | **< 0.0001** |
|  |  | 16 (3.39) | 16 (3.39) | 1 |
|  | Nicotine dependence | 317 (57.74) | 414 (27.22) | **< 0.0001** |
|  |  | 249 (52.75) | 239 (50.64) | 0.51 |
|  | Opioid use disorder | 47 (8.56) | 77 (5.06) | **0.003** |
|  |  | 33 (6.99) | 32 (6.78) | 0.90 |
|  |  |  |  |  |
| For each attribute, upper and lower row represents pre- and post-propensity match data respectively.  To protect patient privacy, TriNetX rounds all counts ≤10 up to 10; as a result, comparisons in which N is 10 should be interpreted with caution.  **Abbreviations**: COPD – chronic obstructive pulmonary disease, IHD – ischemic heart disease, MASLD - Metabolic dysfunction-associated steatotic liver disease, MI – myocardial infarction, NSAID - nonsteroidal anti-inflammatory drugs, NSTEMI – non-ST-segment elevation myocardial infarction, SD – standard deviation, STEMI – ST-segment elevation myocardial infarction. | | | | |

**Supplementary Table 3 –** Baseline characteristics of cirrhosis patients undergoing percutaneous coronary intervention (PCI), stratified by severity of cirrhosis, before and after propensity score matching.

|  | **Characteristic** | | **Decompensated**  **cirrhosis cohort**  **N (%)** | **Compensated cirrhosis**  **N (%)** | ***p*-value** |
| --- | --- | --- | --- | --- | --- |
|  |  | |  |  |  |
| **Demographics** | Age at testing | | Mean 65.1 ± SD 9.0  (N=1,151) | Mean 67.1 ± SD 9.3  (N=2,200) | **< 0.0001** |
|  |  |  | Mean 65.6 ± SD 8.7  (N=980) | Mean 65.6 ± SD 9.3  (N=980) | 0.87 |
|  | Male | | 804 (69.85) | 1,475 (67.05) | 0.09 |
|  |  |  | 676 (68.98) | 688 (70.20) | 0.56 |
|  | White | | 897 (77.93) | 1,705 (77.50) | 0.79 |
|  |  |  | 765 (78.06) | 765 (78.06) | 1 |
|  | African American | | 141 (12.25) | 299 (13.59) | 0.27 |
|  |  |  | 119 (12.14) | 120 (12.24) | 0.94 |
|  | Asian | | 33 (2.87) | 52 (2.36) | 0.43 |
|  |  |  | 29 (2.96) | 26 (2.65) | 0.68 |
|  | Unknown race | | 37 (3.21) | 55 (2.50) | 0.21 |
|  |  |  | 31 (3.16) | 26 (2.65) | 0.50 |
|  | Other race | | 33 (2.87) | 52 (2.36) | 0.43 |
|  |  |  | 26 (2.65) | 33 (3.37) | 0.35 |
| **Body mass index** | | | Mean 29.2 ± SD 6.5  (N=1,021) | Mean 30.4 ± SD 6.8  (N=1,815) | **< 0.0001** |
|  |  |  | Mean 29.1 ± SD 6.5  (N=846) | Mean 30.3 ± SD 7.1  (N=844) | **0.0003** |
| **Medications** | | Antilipemic agents | 994 (86.36) | 2,050 (93.18) | **< 0.0001** |
|  |  |  | 888 (90.61) | 870 (88.78) | 0.18 |
|  |  | Apixaban | 169 (14.69) | 312 (14.18) | 0.73 |
|  |  |  | 150 (15.31) | 149 (15.20) | 0.95 |
|  |  | Beta blockers | 1,062 (92.27) | 1,958 (89.00) | **0.002** |
|  |  |  | 899 (91.73) | 893 (91.12) | 0.63 |
|  |  | Dabigatran | 14 (1.22) | 23 (1.04) | 0.65 |
|  |  |  | 12 (1.22) | 17 (1.73) | 0.35 |
|  |  | Enoxaparin | 575 (49.96) | 1,004 (45.64) | **0.02** |
|  |  |  | 479 (48.88) | 468 (47.75) | 0.62 |
|  |  | Heparin | 1,101 (95.66) | 2,088 (94.91) | **0.34** |
|  |  |  | 936 (95.51) | 930 (94.90) | 0.53 |
|  |  | NSAIDs | 659 (57.25) | 1,386 (63.00) | **0.001** |
|  |  |  | 581 (59.29) | 578 (58.98) | 0.89 |
|  |  | Proton pump inhibitor | 992 (86.19) | 1626 (73.91) | **< 0.0001** |
|  |  |  | 828 (84.49) | 831 (84.80) | 0.85 |
|  |  | Rivaroxaban | 58 (5.04) | 119 (5.41) | 0.65 |
|  |  |  | 51 (5.20) | 52 (5.31) | 0.92 |
|  |  | Warfarin | 147 (12.77) | 312 (14.18) | 0.73 |
|  |  |  | 115 (11.73) | 107 (10.92) | 0.57 |
| **Cardiovascular**  **diseases** | Acute MI | | 633 (55.00) | 1,235 (56.14) | 0.51 |
|  |  |  | 546 (55.71) | 546 (55.71) | 1 |
|  | Atrial fibrillation and flutter | | 310 (26.93) | 626 (28.45) | 0.36 |
|  |  |  | 281 (28.67) | 278 (28.37) | 0.88 |
|  | Cerebrovascular disease | | 392 (34.06) | 725 (32.95) | 0.52 |
|  |  |  | 332 (33.88) | 331 (33.78) | 0.96 |
|  | Chronic IHD | | 1,122 (97.48) | 2,136 (97.09) | 0.47 |
|  |  |  | 954 (97.35) | 955 (97.45) | 0.89 |
|  | Heart failure | | 664 (57.69) | 1,164 (52.91) | **0.008** |
|  |  |  | 557 (56.84) | 556 (56.73) | 0.96 |
|  | Hypertension | | 1,043 (90.62) | 2,047 (93.05) | **.01** |
|  |  |  | 895 (91.33) | 896 (91.43) | 0.94 |
|  | NSTEMI | | 492 (42.75) | 915 (41.59) | 0.25 |
|  |  |  | 419 (42.75) | 418 (42.65) | 0.96 |
|  | Other acute IHD | | 190 (16.51) | 325 (14.77) | 0.18 |
|  |  |  | 153 (15.61) | 152 (15.51) | 0.95 |
|  | Pulmonary embolism | | 69 (5.99) | 88 (4.00) | **.008** |
|  |  |  | 53 (5.41) | 50 (5.10) | 0.76 |
|  | STEMI of anterior wall | | 81 (7.04) | 181 (8.23) | 0.21 |
|  |  |  | 69 (7.04) | 72 (7.35) | 0.79 |
|  | STEMI of inferior wall | | 110 (9.56) | 193 (8.77) | 0.44 |
|  |  |  | 94 (9.59) | 84 (8.57) | 0.43 |
|  | STEMI of other sites | | 36 (3.13) | 72 (3.27) | 0.87 |
|  |  |  | 28 (2.86) | 29 (2.96) | 0.89 |
|  | STEMI of unspecified site | | 198 (17.20) | 372 (16.91) | 0.81 |
|  |  |  | 170 (17.35) | 173 (17.65) | 0.86 |
|  | Unstable angina | | 212 (18.42) | 522 (23.73) | **0.0004** |
|  |  |  | 193 (19.69) | 189 (19.29) | 0.82 |
| **Gastrointestinal and liver diseases** | ^†^Alcohol-associated cirrhosis | | 477 (41.44) | 435 (19.77) | **< 0.0001** |
|  |  |  | 332 (33.88) | 333 (33.98) | 0.96 |
|  | Chronic viral hepatitis | | 307 (26.67) | 664 (30.18) | **0.03** |
|  |  |  | 271 (27.65) | 268 (27.35) | 0.88 |
|  | Fatty liver | | 415 (36.06) | 727 (33.05) | 0.08 |
|  |  |  | 346 (35.31) | 331 (33.78) | 0.48 |
| **Metabolic diseases** | Type 1 diabetes mellitus | | 174 (15.12) | 248 (11.27) | 0.001 |
|  |  |  | 145 (14.80) | 140 (14.29) | 0.75 |
|  | Type 2 diabetes mellitus | | 828 (71.93) | 1,467 (66.68) | **0.002** |
|  |  |  | 695 (70.92) | 694 (70.82) | 0.96 |
|  | Hyperlipidemia | | 975 (84.71) | 1,958 (89.00) | **0.0003** |
|  |  |  | 846 (86.33) | 855 (87.24) | 0.55 |
|  | Hypothyroidism | | 277 (24.07) | 484 (22.00) | 0.17 |
|  |  |  | 233 (23.78) | 222 (22.65) | 0.56 |
|  | Overweight/  obesity | | 597 (51.87) | 1,138 (51.73) | 0.94 |
|  |  |  | 506 (51.63) | 506 (51.63) | 1 |
| **Pulmonary diseases** | COPD | | 344 (29.89) | 754 (34.27) | **0.009** |
|  |  |  | 310 (31.63) | 314 (32.04) | 0.85 |
|  | Dependence on respirator | | 43 (3.74) | 39 (1.77) | **0.0004** |
|  |  |  | 26 (2.65) | 28 (2.86) | 0.78 |
| **Renal diseases** | Chronic kidney disease | | 600 (52.13) | 903 (41.05) | **< 0.0001** |
|  |  |  | 474 (48.37) | 480 (48.98) | 0.79 |
|  | Dependence on renal dialysis | | 150 (13.03) | 134 (6.09) | **< 0.0001** |
|  |  |  | 104 (10.61) | 103 (10.51) | 0.94 |
| **Substance use disorders** | Alcohol use disorder | | 375 (32.58) | 531 (24.14) | **< 0.0001** |
|  |  |  | 293 (29.90) | 284 (28.98) | 0.66 |
|  | Cannabis use disorder | | 94 (8.17) | 170 (7.73) | 0.66 |
|  |  |  | 75 (7.65) | 76 (7.75) | 0.93 |
|  | Cocaine use disorder | | 58 (5.04) | 123 (5.59) | 0.50 |
|  |  |  | 48 (4.90) | 51 (5.20) | 0.76 |
|  | Nicotine dependence | | 452 (39.27) | 910 (41.36) | 0.25 |
|  |  |  | 394 (40.20) | 391 (39.90) | 0.89 |
|  | Opioid use disorder | | 76 (6.60) | 190 (8.64) | **0.04** |
|  |  |  | 72 (7.35) | 75 (7.65) | 0.80 |
|  |  | |  |  |  |
| For each attribute, upper and lower row represents pre- and post-propensity match data respectively.  ^†^For propensity matching, alcohol-associated cirrhosis was defined by ICD-10 code alone, unlike the more stringent cohort-building algorithm used for the cirrhosis etiology subgroup comparison.  **Abbreviations**: COPD – chronic obstructive pulmonary disease, IHD – ischemic heart disease, MI – myocardial infarction, NSAID - nonsteroidal anti-inflammatory drugs, NSTEMI – non-ST-segment elevation myocardial infarction, SD – standard deviation, STEMI – ST-segment elevation myocardial infarction. | | | | | |

**Supplementary Table 4 –** Baseline characteristics of cirrhosis patients undergoing percutaneous coronary intervention (PCI), stratified by antiplatelet monotherapy versus dual therapy after 1 month of PCI, before and after propensity score matching.

|  | **Characteristic** | **Antiplatelet monotherapy**  **N (%)** | **Antiplatelet**  **dual therapy**  **N (%)** | ***p*-value** |
| --- | --- | --- | --- | --- |
|  |  |  |  |  |
| **Demographics** | Age at testing | Mean 66.9 ± SD 9.1  (N=757) | Mean 66.3 ± SD 9.3  (N=1,451) | 0.13 |
|  |  | Mean 66.8 ± SD 9.1  (N=698) | Mean 67.1 ± SD 9.1  (N=698) | 0.52 |
|  | Male | 526 (69.48) | 918 (63.27) | **0.004** |
|  |  | 477 (68.34) | 467 (66.90) | 0.57 |
|  | White | 614 (81.11) | 1,109 (76.43) | **0.01** |
|  |  | 573 (82.09) | 578 (82.81) | 0.72 |
|  | African American | 70 (9.25) | 232 (15.99) | **< 0.0001** |
|  |  | 70 (10.03) | 65 (9.31) | 0.65 |
|  | Asian | 25 (3.30) | 31 (2.14) | 0.10 |
|  |  | 21 (3.01) | 21 (3.01) | 1 |
|  | Unknown race | 24 (3.17) | 22 (1.52) | **.01** |
|  |  | 12 (1.72) | 13 (1.86) | 0.84 |
|  | Other race | 16 (2.11) | 42 (2.89) | 0.28 |
|  |  | 14 (2.01) | 14 (2.01) | 1 |
| **Body mass index** | | Mean 30.0 ± SD 6.3  (N=636) | Mean 30.3 ± SD 6.9  (N=1,212) | 0.43 |
|  |  | Mean 30.0 ± SD 6.3  (N=586) | Mean 30.6 ± SD 6.8  (N=586) | 0.12 |
| **Laboratory parameters** | Creatinine | Mean 1.4 ± SD 1.5  (N=751) | Mean 1.8 ± SD 2.1  (N=1,436) | **< 0.0001** |
|  |  | Mean 1.5 ± SD 1.6  (N=692) | Mean 1.5 ± SD 1.6  (N=692) | 0.89 |
|  | Hemoglobin | Mean 12.5 ± SD 2.4  (N=754) | Mean 11.9 ± SD 2.4  (N=1,442) | **< 0.0001** |
|  |  | Mean 12.5 ± SD 2.5  (N=695) | Mean 12.1 ± SD 2.4  (N=695) | **0.005** |
|  | International normalized ratio | Mean 1.2 ± SD 0.2  (N=718) | Mean 1.2 ± SD 0.2  (N=718) | 0.67 |
|  |  | Mean 1.2 ± SD 0.2  (N=665) | Mean 1.2 ± SD 0.2  (N=670) | 0.80 |
|  | Sodium | Mean 138 ± SD 3.7  (N=755) | Mean 137 ± SD 3.8  (N=1,444) | 0.13 |
|  |  | Mean 137 ± SD 3.7  (N=696) | Mean 137 ± SD 3.9  (N=696) | 0.64 |
|  | Total bilirubin | Mean 0.9 ± SD 0.8  (N=733) | Mean 0.8 ± SD 0.8  (N=1,422) | 0.32 |
|  |  | Mean 0.9 ± SD 0.7  (N=676) | Mean 0.9 ± SD 0.8  (N=677) | 0.67 |
| **Medications** | Antilipemic agents | 684 (90.36) | 1,344 (92.63) | 0.06 |
|  |  | 633 (90.69) | 629 (90.11) | 0.72 |
|  | Apixaban | 133 (17.57) | 202 (13.92) | **0.02** |
|  |  | 116 (16.62) | 116 (16.62) | 1 |
|  | Beta blockers | 675 (89.17) | 1,347 (92.83) | **0.003** |
|  |  | 629 (90.11) | 628 (89.97) | 0.93 |
|  | Dabigatran | 10 (1.32) | 13 (0.90) | 0.35 |
|  |  | 10 (4.33) | 10 (4.33) | 1 |
|  | Enoxaparin | 327 (43.20) | 782 (53.89) | **< 0.0001** |
|  |  | 313 (44.84) | 303 (43.41) | 0.59 |
|  | Heparin | 707 (93.39) | 1,409 (97.10) | **< 0.0001** |
|  |  | 668 (95.70) | 664 (95.13) | 0.61 |
|  | NSAIDs | 452 (59.71) | 968 (66.71) | **.001** |
|  |  | 431 (61.75) | 425 (60.89) | 0.74 |
|  | Proton pump inhibitor | 576 (76.09) | 1207 (83.18) | **< 0.0001** |
|  |  | 541 (77.51) | 536 (76.79) | 0.75 |
|  | Rivaroxaban | 56 (7.40) | 72 (4.96) | **0.02** |
|  |  | 52 (7.45) | 48 (6.88) | 0.68 |
|  | Warfarin | 85 (11.23) | 178 (12.27) | 0.47 |
|  |  | 79 (11.32) | 81 (11.60) | 0.87 |
| **Cardiovascular**  **diseases** | Acute MI | 378 (49.93) | 855 (58.92) | **< 0.0001** |
|  |  | 356 (51.00) | 362 (51.86) | 0.75 |
|  | Atrial fibrillation and flutter | 245 (32.36) | 388 (26.74) | 0.005 |
|  |  | 215 (30.80) | 215 (30.80) | 1 |
|  | Cerebrovascular diseases | 252 (33.29) | 547 (37.60) | **.04** |
|  |  | 240 (34.38) | 238 (34.10) | 0.91 |
|  | Chronic IHD | 737 (97.26) | 1,416 (97.59) | 0.74 |
|  |  | 679 (97.28) | 682 (97.71) | 0.61 |
|  | Heart failure | 366 (48.35) | 867 (59.75) | **< 0.0001** |
|  |  | 348 (49.86) | 351 (50.29) | 0.87 |
|  | Hypertension | 681 (89.96) | 1,383 (95.31) | **< 0.0001** |
|  |  | 639 (91.55) | 647 (92.69) | 0.43 |
|  | NSTEMI | 268 (35.40) | 683 (47.07) | **< 0.0001** |
|  |  | 258 (36.96) | 265 (37.97) | 0.70 |
|  | Other acute IHD | 88 (11.62) | 286 (19.71) | **< 0.0001** |
|  |  | 87 (12.46) | 79 (11.32) | 0.51 |
|  | Pulmonary embolism | 34 (4.49) | 72 (5.03) | 0.58 |
|  |  | 32 (4.58) | 33 (4.73) | 0.90 |
|  | STEMI of anterior wall | 50 (6.60) | 111 (7.65) | 0.37 |
|  |  | 44 (6.30) | 49 (7.02) | 0.59 |
|  | STEMI of inferior wall | 59 (7.79) | 130 (8.96) | 0.35 |
|  |  | 53 (7.59) | 55 (7.88) | 0.84 |
|  | STEMI of other sites | 24 (3.17) | 38 (2.62) | 0.46 |
|  |  | 23 (3.29) | 24 (3.44) | 0.88 |
|  | STEMI of unspecified site | 112 (14.79) | 257 (17.71) | 0.08 |
|  |  | 105 (15.04) | 110 (15.76) | 0.71 |
|  | Unstable angina | 141 (18.63) | 395 (27.22) | **< 0.0001** |
|  |  | 136 (19.48) | 143 (20.49) | 0.64 |
| **Gastrointestinal and liver disease** | ^†^Alcohol-associated cirrhosis | 171 (22.59) | 344 (23.71) | 0.55 |
|  |  | 160 (22.92) | 153 (21.92) | 0.65 |
|  | Chronic viral hepatitis | 214 (28.27) | 443 (30.53) | 0.27 |
|  |  | 202 (28.94) | 202 (28.94) | 1 |
|  | Esophageal varices | 186 (24.58) | 381 (26.26) | 0.40 |
|  |  | 175 (25.07) | 167 (23.93) | 0.65 |
|  | MASH | 192 (25.36) | 387 (26.67) | 0.51 |
|  |  | 181 (25.93) | 177 (25.36) | 0.81 |
|  | Portal hypertension | 254 (33.55) | 505 (34.80) | 0.56 |
|  |  | 239 (34.24) | 234 (33.52) | 0.77 |
| **Metabolic diseases** | Type 1 diabetes mellitus | 81 (10.70) | 233 (16.06) | **0.0006** |
|  |  | 78 (11.17) | 81 (11.60) | 0.80 |
|  | Type 2 diabetes mellitus | 504 (66.58) | 1,081 (74.5) | **< 0.0001** |
|  |  | 470 (67.33) | 485 (69.48) | 0.39 |
|  | Hyperlipidemia | 669 (88.37) | 1,289 (88.83) | 0.75 |
|  |  | 613 (87.82) | 618 (88.54) | 0.68 |
|  | Hypothyroidism | 176 (23.25) | 347 (23.91) | 0.72 |
|  |  | 164 (23.50) | 159 (22.78) | 0.75 |
|  | Overweight/  obesity | 410 (54.16) | 816 (56.24) | 0.35 |
|  |  | 381 (54.58) | 387 (55.44) | 0.75 |
| **Pulmonary diseases** | COPD | 238 (31.44) | 510 (35.15) | 0.08 |
|  |  | 225 (32.23) | 229 (32.81) | 0.82 |
|  | Dependence on respirator | 17 (2.25) | 29 (2.00) | 0.70 |
|  |  | 16 (2.29) | 19 (2.72) | 0.61 |
| **Renal diseases** | Chronic kidney disease | 301 (39.76) | 756 (52.10) | **< 0.0001** |
|  |  | 288 (41.26) | 291 (41.69) | 0.87 |
|  | Dependence on renal dialysis | 46 (6.08) | 170 (11.72) | **< 0.0001** |
|  |  | 46 (6.59) | 41 (5.87) | 0.58 |
| **Substance use disorders** | Alcohol use disorder | 173 (22.85) | 378 (26.05) | 0.10 |
|  |  | 378 (26.05) | 167 (23.93) | 0.90 |
|  | Cannabis use disorder | 60 (7.93) | 115 (7.93) | 1 |
|  |  | 55 (7.88) | 63 (9.03) | 0.44 |
|  | Cocaine use disorder | 31 (4.09) | 86 (5.93) | 0.07 |
|  |  | 30 (4.30) | 32 (4.58) | 0.79 |
|  | Nicotine dependence | 276 (36.46) | 618 (42.59) | **0.005** |
|  |  | 262 (37.54) | 270 (38.68) | 0.66 |
|  | Opioid use disorder | 49 (6.47) | 153 (10.54) | **0.002** |
|  |  | 47 (6.73) | 49 (7.02) | 0.83 |
|  |  |  |  |  |
| For each attribute, upper and lower row represents pre- and post-propensity match data respectively.  To protect patient privacy, TriNetX rounds all counts ≤10 up to 10; as a result, comparisons in which N is 10 should be interpreted with caution.  ^†^For propensity matching, alcohol-associated cirrhosis was defined by ICD-10 code alone, unlike the more stringent cohort-building algorithm used for the cirrhosis etiology subgroup comparison.  **Abbreviations**: COPD – chronic obstructive pulmonary disease, IHD – ischemic heart disease, MASH - metabolic dysfunction-associated steatohepatitis, MI – myocardial infarction, NSAID - nonsteroidal anti-inflammatory drugs, NSTEMI – non-ST-segment elevation myocardial infarction, SD – standard deviation, STEMI – ST-segment elevation myocardial infarction. | | | | |

**Supplementary Table** **5** - Baseline characteristics of cirrhosis patients undergoing percutaneous coronary intervention (PCI), stratified by aspirin versus clopidogrel monotherapy after 1 month of PCI, before and after propensity score matching.

|  | **Characteristic** | **Aspirin monotherapy**  **N (%)** | **Clopidogrel**  **monotherapy**  **N (%)** | ***p*-value** |
| --- | --- | --- | --- | --- |
|  |  |  |  |  |
| **Demographics** | Age at testing | Mean 64.5 ± SD 8.7  (N=224) | Mean 68.1 ± SD 8.3  (N=217) | **< 0.0001** |
|  |  | Mean 66.6 ± SD 7.9  (N=125) | Mean 66.6 ± SD 8.6  (N=125) | 0.99 |
|  | Male | 155 (69.20) | 147 (67.74) | 0.74 |
|  |  | 81 (64.80) | 83 (66.40) | 0.79 |
|  | White | 172 (76.79) | 166 (76.50) | 0.94 |
|  |  | 100 (80.00) | 101 (80.80) | 0.87 |
| **Body mass index** | | Mean 29.6 ± SD 6.0  (N=197) | Mean 30.7 ± SD 6.3  (N=181) | 0.09 |
|  |  | Mean 31.1 ± SD 6.1  (N=108) | Mean 30.1 ± SD 5.4  (N=109) | 0.22 |
| **Laboratory parameters** | Creatinine | Mean 1.6 ± SD 1.7  (N=221) | Mean 1.7 ± SD 1.9  (N=215) | 0.44 |
|  |  | Mean 1.5 ± SD 1.4  (N=124) | Mean 1.8 ± SD 2.1  (N=124) | 0.15 |
|  | Hemoglobin | Mean 12.2 ± SD 2.5  (N=224) | Mean 12.2 ± SD 2.2  (N=215) | 0.99 |
|  |  | Mean 11.9 ± SD 2.3  (N=125) | Mean 12.2 ± SD 2.3  (N=125) | 0.31 |
|  | International normalized ratio | Mean 1.2 ± SD 0.3  (N=221) | Mean 1.2 ± SD 0.2  (N=206) | 0.33 |
|  |  | Mean 1.2 ± SD 0.2  (N=123) | Mean 1.2 ± SD 0.2  (N=120) | 0.17 |
|  | Sodium | Mean 137 ± SD 4.1  (N=224) | Mean 137 ± SD 3.9  (N=215) | 0.26 |
|  |  | Mean 137 ± SD 4.3  (N=125) | Mean 137 ± SD 3.6  (N=125) | 0.15 |
|  | Total bilirubin | Mean 1.1 ± SD 1.1  (N=222) | Mean 0.9 ± SD 0.7  (N=209) | **0.02** |
|  |  | Mean 1.0 ± SD 1.0  (N=676) | Mean 0.9 ± SD 0.8  (N=677) | 0.38 |
| **Medications** | Antilipemic agents | 192 (85.71) | 197 (90.78) | 0.10 |
|  |  | 110 (88.00) | 110 (88.00) | 1 |
|  | Apixaban | 21 (9.37) | 50 (23.04) | **<.0001** |
|  |  | 20 (16.00) | 18 (14.40) | 0.72 |
|  | Beta blockers | 200 (89.29) | 200 (92.17) | 0.30 |
|  |  | 117 (93.60) | 114 (91.20) | 0.47 |
|  | Enoxaparin | 105 (46.87) | 104 (47.93) | 0.82 |
|  |  | 58 (46.40) | 62 (49.60) | 0.61 |
|  | Heparin | 213 (95.09) | 205 (94.47) | 0.77 |
|  |  | 119 (95.20) | 119 (95.20) | 1 |
|  | Proton pump inhibitor | 176 (78.57) | 174 (80.18) | 0.71 |
|  |  | 100 (80.00) | 100 (80.00) | 1 |
|  | Rivaroxaban | 12 (5.36) | 13 (5.99) | 0.77 |
|  |  | 10 (8.00) | 10 (8.00) | 1 |
|  | Warfarin | 22 (9.82) | 34 (15.67) | 0.06 |
|  |  | 17 (13.60) | 16 (12.80) | 0.85 |
| **Cardiovascular**  **diseases** | Acute MI | 125 (55.80) | 102 (47.00) | 0.06 |
|  |  | 60 (48.00) | 64 (51.20) | 0.61 |
|  | Atrial fibrillation and flutter | 55 (24.55) | 86 (39.63) | **0.0007** |
|  |  | 41 (32.80) | 39 (31.20) | 0.79 |
|  | Cerebrovascular diseases | 62 (27.68) | 77 (35.48) | 0.08 |
|  |  | 37 (29.60) | 43 (34.40) | 0.42 |
|  | Chronic IHD | 220 (98.21) | 215 (99.08) | 0.43 |
|  |  | 125 (100.00) | 123 (98.40) | 0.16 |
|  | Heart failure | 107 (47.77) | 128 (58.99) | **0.02** |
|  |  | 65 (52.00) | 63 (50.40) | 0.80 |
|  | Hypertension | 195 (87.05) | 206 (94.93) | **0.004** |
|  |  | 115 (92.00) | 115 (92.00) | 1 |
|  | NSTEMI | 87 (38.84) | 81 (37.33) | 0.74 |
|  |  | 46 (36.80) | 54 (43.20) | 0.30 |
|  | Other acute IHD | 22 (9.82) | 40 (18.43) | **0.009** |
|  |  | 14 (11.2) | 21 (16.80) | 0.20 |
|  | STEMI of anterior wall | 19 (8.48) | 10 (4.61) | 0.10 |
|  |  | 10 (8.00) | 10 (8.00) | 1 |
|  | STEMI of inferior wall | 22 (9.82) | 10 (4.61) | **0.03** |
|  |  | 10 (8.00) | 10 (8.00) | 1 |
|  | STEMI of other sites | 10 (4.46) | 10 (4.61) | 0.94 |
|  |  | 10 (8.00) | 10 (8.00) | 1 |
|  | STEMI of unspecified site | 45 (20.09) | 25 (11.52) | **0.01** |
|  |  | 13 (10.40) | 14 (11.20) | 0.84 |
|  | Unstable angina | 40 (17.86) | 49 (22.58) | 0.22 |
|  |  | 25 (20.00) | 27 (21.60) | 0.75 |
| **Gastrointestinal and liver disease** | ^†^Alcohol-associated cirrhosis | 62 (27.68) | 49 (22.58) | 0.22 |
|  |  | 30 (24.00) | 32 (25.60) | 0.77 |
|  | Chronic viral hepatitis | 65 (29.02) | 57 (26.27) | 0.52 |
|  |  | 31 (24.80) | 32 (25.60) | 0.88 |
|  | Esophageal varices | 49 (21.88) | 49 (22.58) | 0.90 |
|  |  | 27 (21.60) | 28 (23.02) | 0.88 |
|  | MASH | 60 (26.79) | 58 (26.73) | 0.99 |
|  |  | 41 (32.80) | 39 (31.20) | 0.79 |
|  | Portal hypertension | 70 (31.25) | 68 (31.34) | 0.99 |
|  |  | 40 (32.00) | 40 (32.00) | 1 |
| **Metabolic diseases** | Type 1 diabetes mellitus | 19 (8.48) | 27 (12.44) | 0.17 |
|  |  | 12 (9.60) | 13 (10.40) | 0.83 |
|  | Type 2 diabetes mellitus | 142 (63.39) | 145 (66.82) | 0.45 |
|  |  | 84 (67.20) | 83 (66.40) | 0.89 |
|  | Hyperlipidemia | 184 (82.14) | 196 (90.32) | **0.01** |
|  |  | 111 (88.80) | 109 (87.20) | 0.70 |
|  | Overweight/  obesity | 118 (52.68) | 128 (58.99) | 0.18 |
|  |  | 78 (62.40) | 73 (58.40) | 0.52 |
| **Renal diseases** | Chronic kidney disease | 87 (38.84) | 108 (49.77) | **0.02** |
|  |  | 53 (42.40) | 58 (46.40) | 0.52 |
|  | Dependence on renal dialysis | 20 (8.93) | 17 (7.83) | 0.68 |
|  |  | 10 (8.00) | 14 (11.20) | 0.39 |
| **Substance use disorders** | Alcohol use disorder | 59 (26.34) | 47 (21.66) | 0.25 |
|  |  | 28 (22.4) | 29 (23.20) | 0.88 |
|  | Nicotine dependence | 94 (41.96) | 69 (31.80) | **0.03** |
|  |  | 40 (32.00) | 45 (36.00) | 0.50 |
|  | Opioid use disorder | 21 (9.37) | 14 (6.45) | 0.26 |
|  |  | 10 (8.00) | 10 (8.00) | 1 |
|  |  |  |  |  |
| For each attribute, upper and lower row represents pre- and post-propensity match data respectively.  To protect patient privacy, TriNetX rounds all counts ≤10 up to 10; as a result, comparisons in which N is 10 should be interpreted with caution.  ^†^For propensity matching, alcohol-associated cirrhosis was defined by ICD-10 code alone, unlike the more stringent cohort-building algorithm used for the cirrhosis etiology subgroup comparison.  **Abbreviations**: COPD – chronic obstructive pulmonary disease, IHD – ischemic heart disease, MASH - metabolic dysfunction-associated steatohepatitis, MI – myocardial infarction, NSAID - nonsteroidal anti-inflammatory drugs, NSTEMI – non-ST-segment elevation myocardial infarction, SD – standard deviation, STEMI – ST-segment elevation myocardial infarction. | | | | |

**Supplementary Figure -** Kaplan–Meier curves depicting the 1-year risk of mortality among patients undergoing percutaneous coronary intervention (PCI) in the primary cohort and subgroup analyses: (A) cirrhosis versus non-cirrhosis patients, (B) cirrhosis etiology, (C) compensated versus decompensated cirrhosis, (D) antiplatelet monotherapy versus dual antiplatelet therapy, and (E) aspirin versus clopidogrel monotherapy.
